# SIRT1, a novel transcriptional downstream target of CD44, linking its deacetylase activity to tumor cell invasion/metastasis

**DOI:** 10.3389/fonc.2022.1038121

**Published:** 2022-11-23

**Authors:** Salma M. S. Ahmad, Maryam Al-Mansoob, Allal Ouhtit

**Affiliations:** Biological Sciences Program, Department of Biological & Environmental Sciences, College of Arts and Science, Qatar University, Doha, Qatar

**Keywords:** *SIRT1*, breast cancer, CD44, hyaluronan, metastasis

## Abstract

Our tetracycline-off-inducible CD44 expression system previously established in mouse model, revealed that activation of CD44 with its major ligand hyaluronan (HA) promoted breast cancer (BC) metastasis to the liver. To identify the mechanisms that underpin CD44-promoted BC cell invasion, microarray gene expression profiling using RNA samples from (Tet)-Off-regulated expression system of CD44s in MCF7 cells, revealed a set of upregulated genes including, nuclear sirtuin-1 (*SIRT1* also known as NAD-dependent deacetylase), an enzyme that requires NAD^+^ as a cofactor to deacetylate several histones and transcription factors. It stimulates various oncogenic pathways promoting tumorigenesis. This data suggests that *SIRT1* is a potential novel transcriptional target of CD44-downstream signaling that promote BC cell invasion/metastasis. This review will discuss the evidence supporting this hypothesis as well as the mechanisms linking *SIRT1* to cell proliferation and invasion.

## Background

Breast Cancer (BC) is the most common malignant tumor in women worldwide including the state of Qatar, which may arise either as a result of family history or exposure to harmful environmental factors such as radiation, high alcohol consumption, and lifestyle (1–3). Unfortunately, malignant tumors has the capability to metastasize, which involves both migration and invasion of cancer cells (4). The process of metastasis begins when cancerous cells detach from the primary tumor found in a specific organ or tissue and start invading through the extracellular matrix to the blood vessels. Cancerous cells will keep circulating in blood vessels unless it is detected by immune cells for degradation or until it finds a suitable organ with a good blood supply to invade into, forming a secondary tumor (5). The process of invasion encompasses three major components including, cell adhesion molecules (CAMs) (6, 7) found on the cell surface to help invading cells adhere to the surrounding extracellular matrix (ECM) (8).

Since CAMs play a vital role in invasion, our own work has concentrated since 2006 on a CAM protein family known as CD44 (9–14). CD44 is a cell surface receptor for its main ligand hyaluronic acid (HA) (15, 16) which stimulates various oncogenic signaling pathways (e.g. Rho GTPases, PI3K/AKT, and MAPK signaling pathways) resulting in tumor cell survival, proliferation, migration and invasion (17). A better understanding of the various CD44-downstream mechanisms promoting metastasis will ultimately help in developing effective anti-metastatic therapeutic strategies. Consequently, to further investigate CD44 mechanisms associated with the process of invasion, we have previously developed a tetracycline (Tet)-Off-regulated expression system of CD44 in both *in vitro* (9) and *in vivo* (18). A microarray analysis was further carried out to identify CD44-transcriptional target genes. Based on the microarray results we have previously validated three target genes along with their signaling pathways (Cortactin, Survivin and TGF-β2) as novel downstream target genes that underpin CD44-promoted breast tumor cell invasion (9, 10, 19). From the same microarray data, *Sirtuin 1 (SIRT1)*, was selected for further validation studies as potential target of CD44 because of its involvement in cell proliferation, invasion, and metastasis.

*SIRT1* is one of the seven members of the Sirtuins family belonging to the third class of histone deacetylase enzymes, that require a significant co-factor known as nicotinamide adenine dinucleotide (NAD^+^) (20). Nuclear *SIRT1* was reported to catalyze the deacetylation of lysin residues found within histone proteins including H1, H3, and H4; It deacetylates several oncogenes and transcription factors thereby affecting their function (21, 22). Furthermore, recent studies demonstrated that cytoplasmic *SIRT1* plays a significant role in cell proliferation, cell cycle, apoptosis, energy metabolism, and DNA repair, suggesting that *SIRT1* plays a key role in tumorigenesis, development, and drug resistance (21). This review focuses on discussing the literature data supporting *SIRT1* as a potential novel target of CD44-downstream signaling underlying the process of BC cell invasion.

## Structure of SIRT1

*SIRT1* is encoded by a gene located on the long arm of chromosome 10 (10q21.3), that is composed of 747 amino acids forming four regions, the nuclear localization signal 41-46 amino acids found at the N-terminal, the allosteric side located from 184 to 243 amino acid, the preserved catalytic domain, where deacetylation occur at the centre of the domain, and the C-terminal region located from 631 to 365 amino acid. The N-terminal region is significant as it is where the nuclear reading occurs allowing *SIRT1* to translocate to the nucleus (23, 24).

The catalytic domain is composed of 277 residues consisting of a larger NAD^+^-binding subdomain containing a Rossmann-fold, and a smaller subdomain that is created by two insertions in the NAD^+^-binding domain: i) a helical module (residues 269 to 324) and ii) a Zn^2+^-binding module (residues 362-419). The NAD^+^-binding domain consists of six-stranded parallel β sheets and eight α helices. However, the Zn^2+^-binding domain is composed of 3 β strands and a single α helices. *SIRT1* C-terminal regulatory segment was found to form a quaternary complex with NAD^+^-binding domain, by binding to its lower edge to match the central parallel β sheet of its Rossmann fold (25). *SIRT1* transfers the acetyl group from ε-N-acetyl lysine amino acids on the histones that wraps the DNA, controlling the transcription of genes in a NAD^+^-dependant manner (24).

## Functions of SIRT1

Physiologically, *SIRT1* is expressed in both normal and malignant cells. The following sections will discuss the role of *SIRT1* in both normal and malignant cells.

### Physiological functions of SIRT1 in normal cells

*SIRT1* was found to be upregulated in the body during fasting and calorie restriction, as it is a key regulator of metabolism. Its overexpression controls mitochondrial biogenesis, stimulating the catabolism of fat and cholesterol found in the liver, skeletal muscle, and adipose tissues. Moreover, upregulation of *SIRT1* will induce the expression of gluconeogenic gene, the activation of fatty acid oxidation and the suppression of glycolytic genes, by controlling the transcription of PGC-1α (20). Moreover, *SIRT1* doesn’t only coordinate with PGC-1α, but also enhances the expression of SIRT6 and SIRT5. SIRT6 enhances the production of metabolic intermediates by regulating the mitochondrial activity. On the other hand, SIRT5 is involved in the apoptotic pathways, as it deacetylates cytochrome c (20). Furthermore, *SIRT1* is also found to be expressed in pro-opiomelanocortin neurons, which are significant in controlling normal body weight by regulating glucose homeostasis. The knock-down of *SIRT1* in these neurons causes hypersensitivity and anterior pituitary cell defects failing to regulate changes in pituitary signaling (20, 26). In addition, *SIRT1* acts as a positive regulator monitoring insulin secreted by the pancreas. The presence of *SIRT1* enhances glucose tolerance in pancreatic β-cells by improving the process of insulin secretion. Nevertheless, suppression of *SIRT1* damages insulin secretion process stimulated by glucose (26).

### Functions of SIRT1 in cancer cells and its association with CD44

Previous studies have stated that *SIRT1* expression was overexpressed in BC compared to its expression in normal cells. Overexpressing *SIRT1* in MCF-7 cells has promoted their proliferation, migration, and invasion (21). Moreover, *SIRT1* has an oncogenic activity in BC cells as it inhibits the expression of the tumour suppressor gene p53 *via* activation of Mdm2, interfering with cell proliferation, cell cycle, apoptosis, and DNA repair, predisposing breast cells to neoplastic transformation (21, 27). Further studies have shown that *SIRT1* is upregulated in BC cells, promoting cell proliferation and cell cycle progression through its interaction with PI3K/AKT oncogenic pathway (28). Likewise, silencing *SIRT1* inhibited the activation of PI3K/AKT pathway (29). On one hand, we have previously demonstrated that CD44 activates PI3K/AKT pathway to promote cellular migration, invasion, and survival (12, 13). On the other hand, PI3K/AKT activates *SIRT1* (30), all this data put together suggest that CD44 might activate *SIRT1 via* PI3K/AKT.

Cytoplasmic *SIRT1* directly interact with MAPK/Ras/ERK pathways promoting neuronal differentiation and survival. Furthermore, the suppression of *SIRT1* decreased the phosphorylation of JNK/ERK/MAPK signalling pathways in cerebral ischemia in both rats and humans (31). Similarly, CD44 activates ERK phosphorylation, activating both extracellular and intracellular signals to promote cell proliferation and migration (32). In addition, CD44 also phosphorylates ERK/MAPK and RAS/MAPK signalling pathway to promote tumour angiogenesis, migration, and invasion (12, 33, 34).

**Figure 1:**
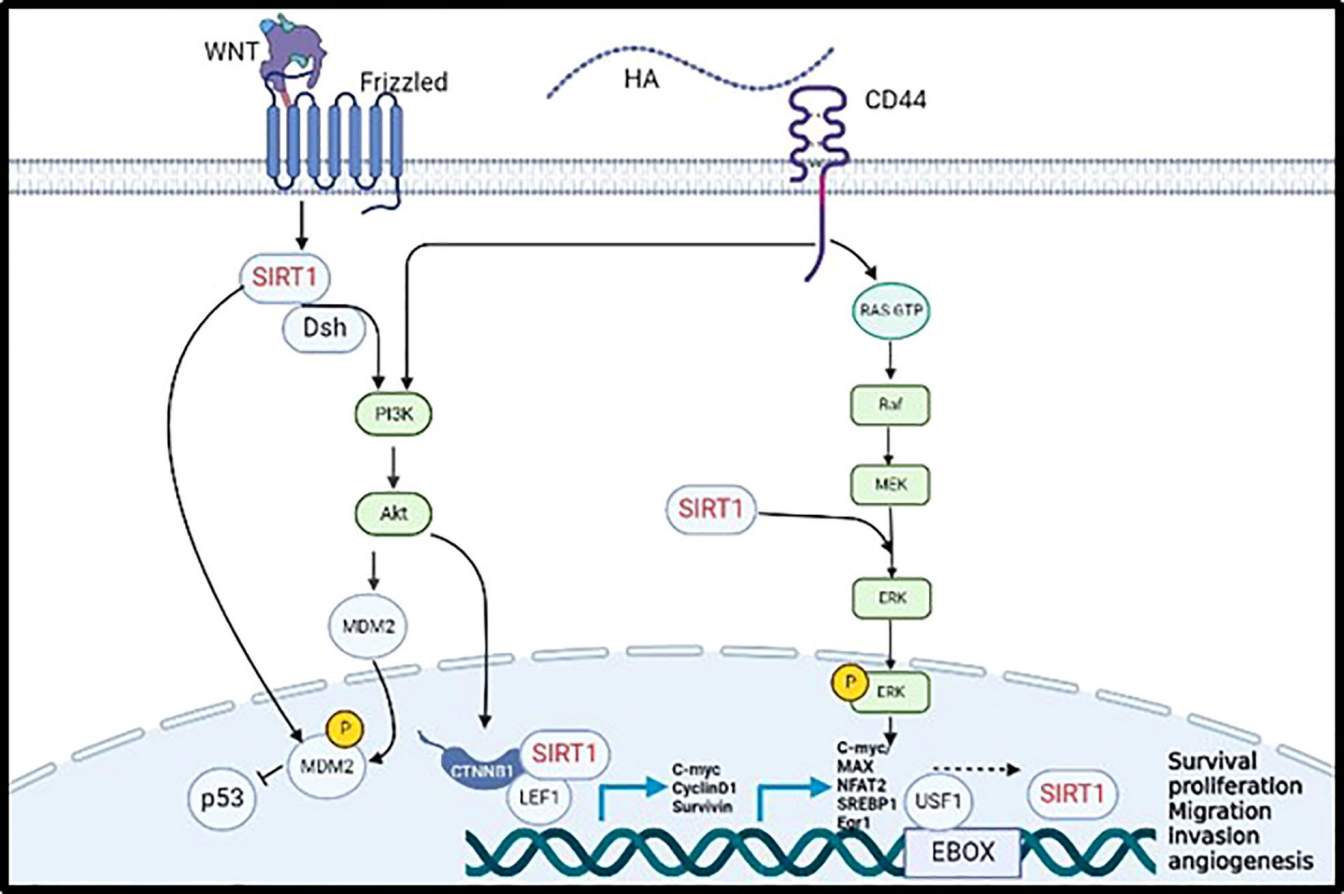
Validated (→) and proposed (– – – →) mechanisms linking CD44 activation by hyaluronan to induce SIRT1 transcription promoting tumor cell progression and metastasis.

Cytoplasmic *SIRT1* was also found to directly interact with cytoplasmic cortactin promoting cell migration, invasion, and metastasis, through IGF-1 activation in non-small cell lung cancer. Several studies stated that cortactin was upregulated in various human cancers such as breast, head, oesophagus, and hepatic cancers (35). Our previous studies showed that CD44 activates cortactin *via* the transcription factor NF-κB (9); This data suggest that CD44 might activate *SIRT1 via* activation of cortactin or its associated signalling pathways. *SIRT1* also plays a significant role in activating Wnt signaling acting as tumour promoter in colorectal cancer. First, Adenomatous polyposis coli (APC) regulate Wnt Signaling pathway by translocating β-catenin from the cytoplasm into the nucleus activating several oncogenic pathways. The cytoplasmic *SIRT1* colocalizes dsh protein in the cytoplasm and enhances the expression of DNA Methyl-transferase 1 (DNMT1) that promote DNA hypermethylation in the promoter domain of APC, thereby inhibiting its tumour suppressor function. The Dsh protein will inhibit the phosphorylation of β-catenin allowing its accumulation in the nucleus, upregulating the transcription factors TCF/LEF (T-cell factor/Lymphoid enhancer factor). Nuclear *SIRT1* will bind to the LEF1 lysine residue to deacetylate the present histones regulating the transcription of the downstream targets such as cyclin D1, C-Myc, and surviving, thereby inducing tumour proliferation and migration (24). Moreover, the activation of Wnt signaling activates *SIRT1* to interact with Dsh forming a complex that will phosphorylate and activate PI3K/AKT signaling pathway, that will also result in the translocation of β-catenin into the nucleus to activate its downstream targets promoting cell migration in colon and BC cells (24, 36, 37). Correspondingly, the activation of Wnt/β-catenin pathway will activate the direct interaction of CD44 with cortactin and will enhance the transcription of CD44 and c-myc in indicating positive feedback of Wnt signaling-CD44 loop promoting cell adhesion, migration, and invasion of BC and melanoma (9, 38). In addition, overexpression of CD44 upregulates the expression of cyclin D1 through the activation of ERK pathway that will promote tumour proliferation and migration of BC, ovarian cancer, and squamous cell carcinoma (32). Our previous study has proven that activated HA/CD44 has activated PI3K signalling pathway to phosphorylate the transcription factor E2F1 promoting the expression of Survivin, resulting in breast tumour invasion (10). Furthermore, using bioinformatics tools, various transcription factors were identified including, C-Myc/Max, NFAT2, SREBP1, EGR-1 and USF1, that can bind the promoter of *SIRT1 via* induction of MAPK/ERK and PI3K/AKT signalling pathways as shown in **Figure 1** (39).

In summary and as shown in **Figure 1**, SIRT1 is activated by the WNT cell surface transmembrane receptor known as frizzled to inhibit the expression of P53 through MDM2 phosphorylation, either by direct phosphorylation of MDM2 or by activating PI3K/AKT pathway. Moreover, CD44 activates PI3K//AKT pathway allowing the translocation of SIRT1 to the nucleus and its binding to β-catenin and LEF1 to transcribe C-myc, Cyclin D1, and Survivin. Furthermore, CD44 activate the MAPK/ERK pathway which enhance the transcription of SIRT1 by activating C-myc, MAX, NFAT2, SREBP1, Egr1 and USF1 transcription factors. Put together, all the evidence collected from the literature support our hypothesis that *SIRT1* is a novel downstream transcriptional target of CD44/HA regulating pro-metastatic signalling pathways that are involved in tumour proliferation, migration, and invasion.

## Potential therapeutic strategies targeting *SIRT1*

Several studies have been performed to develop suitable inhibitors targeting *SIRT1* to guide the design of applicable therapeutic strategies against BC. Splitomicin, Sirtinol and ILS-JGB-1741 are the inhibitor drugs used to inhibit the expression of *SIRT1* in BC cells, which have been shown to inhibit cell proliferation, induce cell cycle arrest and apoptosis (20, 24).

## Conclusion

*SIRT1* shows a significant role in the development and metastasis of breast tumours, but its underlying mechanisms are still poorly understood. *SIRT1* interferes with various signalling pathways that promote breast tumour cell proliferation, migration, and invasion. *SIRT1* inhibits the expression of P53, interfering with apoptosis leading to survival and tumor cell proliferation. To summarize, it is clear that CD44 activates *SIRT1* most likely *via* two intermediate players PI3K/AKT and MAPK/ERK signalling pathways. These findings support our hypothesis suggesting that *SIRT1* is a novel downstream target that underpin CD44/HA enhancing tumour cell development and metastasis.

## Author contributions

SMSA: writing—original draft (lead), MA-M: Editing, AO: Conceptualization (lead), funding acquisition (lead), writing-review and editing. All authors contributed to the article and approved the submitted version.

## Funding

This research was funded by Qatar University Internal grant number: QUST-1-CAS2019-22 and the Qatar Foundation grant number: UREP24-117-1-027.

## Conflict of interest

The authors declare that the research was conducted in the absence of any commercial or financial relationships that could be construed as a potential conflict of interest.

## Publisher’s note

All claims expressed in this article are solely those of the authors and do not necessarily represent those of their affiliated organizations, or those of the publisher, the editors and the reviewers. Any product that may be evaluated in this article, or claim that may be made by its manufacturer, is not guaranteed or endorsed by the publisher.

## Abbreviations

APC, Adenomatous polyposis coli; AKT, Protein kinase B; BC, Breast cancer; CAM, Cell adhesion molecule; CD44, Cluster of differentiation 44; DNMT1, DNA Methyltransferase 1; ECM, Extracellular matrix; EMT, Epithelial-mesenchymal transition; ERK, Extracellular-signal-regulated kinase; HA, Hyaluronic acid; NAD, Nicotinamide Adenine Dinucleotide; SIRT1, Sirtuin1; PI3K, phosphoinositide 3-kinase; Tet, Tetracycline; TGF-β2, Transforming growth factor-beta 2.
